# *Mycobacterium tuberculosis* infection drives osteoclast overactivation via α2,3-Sialylation to promote pathological bone destruction

**DOI:** 10.3389/fphar.2026.1738896

**Published:** 2026-04-02

**Authors:** Zhiwei Jiang, Ziyang Zhang, Dongyang Zhang, Qixiu Yu, Jiezhong Deng, Ying Qu, Yusheng Yang, Zehua Zhang, Shuquan Guo, Jie Zhang, Ce Dou, Fei Luo

**Affiliations:** 1 Department of Orthopedics, Southwest Hospital, Army Medical University, Chongqing, China; 2 Department of Orthopaedics, The First Affiliated Hospital of Chongqing Medical University, Chongqing, China

**Keywords:** bone destruction, glycerophospholipid metabolism, *Mycobacterium tuberculosis*, osteoclast, α2,3-sialylation

## Abstract

**Introduction:**

Bone tuberculosis is characterized by severe bone destruction driven by aberrant osteoclast overactivation. However, the direct mechanism by which Mycobacterium tuberculosis (Mtb) mediates this pathological process remains unclear. Understanding the molecular basis of pathogen-driven osteoclast dysregulation is essential for developing effective host-directed therapeutic strategies.

**Methods:**

Transcriptomic profiling was performed to identify differentially expressed sialylation-related genes and activated signaling pathways in Mtb-infected cells. Murine bone-tuberculosis models and in vitro osteoclast cultures were employed to assess osteoclast activity and surface α2,3-sialylation levels following Mtb infection. Functional interventions included enzymatic removal of α2,3-sialic acid and pharmacological inhibition of ST3GAL1. Metabolomic analysis was conducted to characterize Mtb-induced alterations in glycerophospholipid metabolism.

**Results:**

Transcriptomic profiling revealed upregulation of sialylation-related genes and activation of TLR2-dependent signaling upon Mtb infection, providing a molecular basis for pathogen-driven surface glycan modifications. In both murine bone-tuberculosis models and in vitro osteoclast cultures, Mtb infection concurrently enhanced osteoclast activity and surface α2,3-sialylation. Enzymatic desialylation or ST3GAL1 inhibition markedly attenuated this overactivation. Metabolomic analysis further demonstrated Mtb-induced reprogramming of glycerophospholipid metabolism, potentially supplying substrates for sialylated glycoconjugate biosynthesis.

**Discussion:**

These findings identify α2,3-sialylation as a central driver of Mtb-induced pathological osteoclast activity, mechanistically linking TLR2 signaling, surface glycan remodeling, and metabolic reprogramming. The coordinate regulation of membrane glycoconjugate biosynthesis and glycerophospholipid metabolism suggests an integrated host response exploited by Mtb to promote bone destruction. Collectively, host glycosylation machinery and associated metabolic pathways represent promising targets for host-directed therapy in bone tuberculosis.

## Introduction

Skeletal homeostasis is maintained by a finely tuned balance of bone remodeling, a lifelong process coordinated by the coupling of bone resorption and formation (Salhotra et al., 2020; Veis and O’Brien, 2023; Tuckermann and Adams, 2021). Osteoclasts, the only terminally differentiated cells specialized for bone resorption, arise from monocyte–macrophage lineage precursors and fuse under RANKL–RANK–OPG signaling to form multinucleated mature cells capable of degrading bone matrix through proton and proteolytic enzyme secretion. Their differentiation and resorptive activity are tightly regulated by molecular cues including macrophage colony-stimulating factor (M-CSF) and the NF-κB/NFATc1 pathway, ensuring spatial and temporal coordination of bone turnover. Disruption of these regulatory networks leads to pathological remodeling; for instance, excessive osteoclast activity drives trabecular bone loss in osteoporosis, whereas impaired osteoclast function results in increased bone mass in osteopetrosis (Stegen and Carmeliet, 2024; Liu et al., 2024). In infectious bone diseases, particularly under chronic inflammation or microbial colonization, sustained metabolic imbalance is commonly observed, with pathogens aberrantly activating osteoclasts via diverse molecular mechanisms, ultimately promoting progressive bone loss (Wang et al., 2022; Yang et al., 2025; Pokhrel et al., 2024). These observations establish osteoclasts as central regulators of osteolytic pathology, acting not only as primary effectors of resorption but also as interpreters of pathological signals, highlighting their relevance for mechanistic exploration and therapeutic targeting.

Cell surface glycosylation, particularly α2,3-sialylation, has emerged as a critical molecular switch modulating osteoclast function (Yang et al., 2023). Sialic acids (SAs), predominantly N-acetylneuraminic acid (Neu5Ac), are terminal residues on glycoconjugates, whose dynamic modifications are catalyzed by sialyltransferases. These modifications reshape cell surface receptor conformation and influence cell–cell recognition and signal transduction (Xie et al., 2021; Chen et al., 2025). Notably, ST3 β-galactoside α2,3-sialyltransferase 1 (ST3GAL1)–mediated α2,3-sialylation selectively enhances Toll-like receptor 2 (TLR2) sialylation, facilitating interaction with Siglec-15 and activating the DAP12/Syk axis to drive osteoclast precursor fusion (Dou et al., 2022). Pathological amplification of ST3GAL1-mediated α2,3-sialylation has been observed under diverse disease conditions. In bone marrow, hyperactive sialylation signaling not only promotes tumor bone metastasis but also accelerates osteolytic activity (Boelaars and Van Kooyk, 2024; Rodriguez et al., 2021; Van De Wall et al., 2020). Beyond receptor modulation, emerging evidence suggests that glycosylation, including α2,3-SA, may interact with cellular metabolic pathways to support membrane remodeling and energy homeostasis, linking post-translational modifications with intracellular metabolic states. This raises the possibility that infection-driven changes in sialylation may coordinate osteoclast metabolism to promote activation.

Bone tuberculosis (TB), comprising roughly 10%–15% of extrapulmonary TB cases, is one of the most prevalent manifestations outside the lungs. Although its mortality is lower than pulmonary TB, bone TB induces progressive and irreversible bone destruction, leading to vertebral collapse, joint deformities, and neurological complications, causing significant disability and socioeconomic burden worldwide (Dartois and Rubin, 2022; Pandita et al., 2020). Aberrant osteoclast activation is recognized as a central driver of bone pathology in bone TB. Mtb can indirectly promote osteoclast overactivation by stimulating immune cells to release proinflammatory cytokines such as TNF-α and IL-6 or by perturbing the osteoblast-mediated OPG/RANKL balance (Liu et al., 2020; MohanaSundaram et al., 2024; Saito et al., 2004). However, direct effects of Mtb on osteoclasts and the underlying molecular mechanisms remain insufficiently characterized. Recent studies indicate that Mtb can modulate host cell surface glycosylation, including sialylation pathways, to influence immune evasion and granuloma formation (Matos et al., 2021). Given the pivotal role of ST3GAL1-mediated α2,3-sialylation in osteoclast fusion and activity, it remains unclear whether Mtb similarly regulates this glycosylation in osteoclasts within bone TB lesions, contributing to pathological bone resorption. Clarifying this mechanism constitutes the primary objective of the present study, and offers a rationale for investigating associated metabolic rewiring as a permissive factor for enhanced α2,3-sialylation and osteoclast activation.

This study provides mechanistic insight into the role of ST3GAL1-mediated α2,3-sialylation in *Mycobacterium tuberculosis*–associated osteoclast activation and consequent pathological bone resorption. Using both a murine calvarial infection model and *in vitro* assays, we establish α2,3-sialylation as a critical post-translational modification required for osteoclast responsiveness to Mtb. Enzymatic removal of sialic acids mitigates osteoclast hyperactivation, underscoring its therapeutic potential in bone tuberculosis. Untargeted metabolomics further implicates infection-induced glycerophospholipid reprogramming as a metabolic basis for enhanced α2,3-sialylation, providing a testable hypothesis for future investigation. Collectively, these findings advance mechanistic understanding of tuberculous bone pathology and support precision strategies targeting sialylation pathways.

## Methods

### Key resources table

**Table:** 

Reagent or resource	Source	Identifier
Antibodies		
Rabbit anti-ST3GAL1 Ab	ABclonal	Cat# A11616 RRID:AB_2758638
Rabbit anti-GAPDH Ab	Servicebio	Cat# ZB15004-HRP-100
Goat anti-rabbit IgG (H + L) HRP	Affinity biosciences	Cat# S0001 RRID:AB_2839429
Biotin MAL-II	Vector laboratories	Cat# B-1265-1
FITC-streptavidin	Biolegend	Cat# 405201
Bacterial and virus strains		
*Mycobacterium bovis* BCG pasteur 1173P2	ATCC	ATCC 35734
Chemicals, peptides, and recombinant proteins		
Bovine serum albumin	Sigma-aldrich	Cat# A2153
Recombinant mouse M-CSF protein	R&D systems	Cat# 416-ML
Recombinant mouse RANKL protein	R&D systems	Cat# 462-TEC
Sialidase (SialEXO23 specifically hydrolyzes α2,3-sialic acids)	Genovis	Cat# G1-SD2-005
0.25% Trypsin	Servicebio	Cat# G4004
PBS	Servicebio	Cat# G4202
Phalloidin	ABclonal	Cat# RM02835
RIPA lysis buffer	Beyotime biotechnology	Cat# P0013B
Protease inhibitors	Beyotime biotechnology	Cat# P1045
DAPI	Beyotime biotechnology	Cat# C1006
2X SG fast qPCR master mix	Sangon biotech	Cat# B639271
Critical commercial assays		
TRAP staining kit	Servicebio	Cat# G1050
RNA-quick purification kit	YiShanBiotech	Cat# ES-RN001
Experimental models: Organisms/strains		
Mouse: C57BL/6J	Southwest hospital animal center	N/A
Oligonucleotides		
siRNA targeting sequence for *St3gal1*, 1# CCA​GGA​CAA​GGU​AUC​AUA​UTT (*F*), AUA​UGA​UAC​CUU​GUC​CUG​GTT (*R*), 2# GCU​GUG​CAG​UUG​UAG​GAA​ATT (*F*), UUU​CCU​ACA​ACU​GCA​CAG​CTT (*R*), 3# GCA​CCA​UCA​CUC​ACA​CCU​ATT (*F*), UAG​GUG​UGA​GUG​AUG​GUG​CTT (*R*)	This paper	N/A
Primers for *St3gal1* CATCAGCCAGGACAAGGTATCAT *(F)*, CCACCAGCCTCTTGTTCAACATA *(R)*	This paper	N/A
Primers for *Nfatc1* ACCAGCGTTTCACGTACCTT *(F)*, AACCTCCTCTCAGCTCACTCT *(R)*	This paper	N/A
Primers for *Fos* TACTACCATTCCCCAGCCGA *(F)*, GCTGTCACCGTGGGGATAAA *(R)*	This paper	N/A
Primers for *Mmp9* CAGCCGACTTTTGTGGTCTTC *(F)* CCCGGGTGTAACCATAGCG *(R)*	This paper	N/A
Primers for *Ocstamp* CGGTAACTGGACGTTTGGGA *(F)* AAATTGGAGGAGTGCCGAGG *(R)*	This paper	N/A
Primers for *Ctsk* GCACCCTTAGTCTTCCGCTC *(F)*, ACCCACATCCTGCTGTTGAG *(R)*	This paper	N/A
Primers for Gapdh GGTTGTCTCCTGCGACTTCA *(F)*, TGGTCCAGGGTTTCTTACTCC *(R)*	This paper	N/A
Software and algorithms		
Adobe illustrator	N/A	https://www.adobe.com/
Adobe photoshop	N/A	https://www.adobe.com/
GraphPad prism 8.0	N/A	https://www.graphpad.com/
ImageJ v1.8.0	N/A	https://imagej.net/
CTvox v3.3.0	N/A	https://www.bruker.com/en/products-andsolutions/diffractometers-and-x-raymicroscopes/3d-x-ray-microscopes/xrm-software.html
DataViewer v1.5.6.2	N/A	https://www.bruker.com/en/products-andsolutions/diffractometers-and-x-raymicroscopes/3d-x-ray-microscopes/xrm-software.html
CTAN v1.20.3.0	N/A	https://www.bruker.com/en/products-and-solutions/diffractometers-and-x-ray-microscopes/3d-x-raymicroscopes/xrm-software.html

## Experimental model and study participant details

### Mice

C57BL/6J male mice (6–8 weeks old) were obtained from the Southwest Hospital Animal Facility and housed under specific pathogen–free conditions with a 12 h light/dark cycle and unrestricted access to food and water. All animal procedures were approved by the Institutional Animal Care and Use Committee of the Army Medical University and conducted in accordance with institutional and national guidelines for laboratory animal welfare.

### Primary cell culture and osteoclastogenesis

Bone marrow cells were isolated from the femora and tibiae of 6–8-week-old male C57BL/6J mice (Southwest Hospital Animal Center). Freshly isolated marrow cells were cultured in α-Minimum Essential Medium (α-MEM; Gibco) supplemented with 10% fetal bovine serum (FBS; Gibco), 1% penicillin–streptomycin (Gibco), and macrophage colony-stimulating factor (M-CSF, 50 ng/mL; R&D Systems) for 48 h to generate bone marrow–derived macrophages (BMMs). To induce osteoclastogenesis, BMMs were subsequently treated with receptor activator of nuclear factor κB ligand (RANKL, 50 ng/mL; R&D Systems) in α-MEM containing 10% FBS. Pre-osteoclasts (pOCs) were collected after 2 days of RANKL and M-CSF exposure, and multinucleated mature osteoclasts (mOCs) were obtained after 5 days of continuous stimulation. All cultures were maintained at 37 °C in a humidified 5% CO_2_ incubator. *Mycoplasma* contamination was routinely excluded, and only primary, non-passaged cells were used to preserve native biological properties.

## Method details

### Murine calvarial tuberculosis model

Male C57BL/6J mice (six to eight weeks old) were randomly allocated into three groups: vehicle control (PBS), *M. bovis* BCG infection (1 × 10^6^ CFU per mouse), and BCG combined with sialidase treatment (10 U SialEXO23, a selective α2,3-sialidase). Subcutaneous injections were administered over the calvarial surface under anesthesia every other day for a total of seven administrations. Mice were euthanized 48 h after the final (seventh) injection. Following euthanasia, calvarial samples were harvested and subjected to micro–computed tomography (μCT) using a Skyscan 1272 system (Bruker, Belgium), and 3D reconstructions were performed with CTvox software. Subsequently, samples were decalcified in EDTA for 2 weeks, processed for paraffin embedding, and sectioned for hematoxylin and eosin (H&E) and tartrate-resistant acid phosphatase (TRAP) staining following manufacturer instructions. Histological evaluation was performed by light microscopy.

### Cell infection

*Mycobacterium bovis* BCG was cultured in Middlebrook 7H9 medium supplemented with 10% OADC and 0.05% Tween-80 to mid-log phase (OD_600_ = 0.8–1.0), pelleted, resuspended in antibiotic-free α-MEM, and filtered through a 5-µm syringe filter to obtain single-cell suspensions (MOI = 10). Bone marrow–derived macrophages were differentiated with M-CSF and RANKL, and on day 2 of RANKL induction, cells were infected with BCG under antibiotic-free conditions. Where indicated, pre-treatments included incubation with α2,3-specific sialidase (0.1 U/mL, 1 h, 37 °C, serum-free medium) or transfection with siRNAs targeting *St3gal1*. Infected and control osteoclasts were subsequently collected for transcriptomic profiling, metabolomic analysis, and additional downstream experiments. To evaluate the viable intracellular bacterial load, infected cells were washed with PBS and lysed using 0.1% Triton X-100 in PBS. The resulting cell lysates were serially diluted and plated onto Middlebrook 7H10 agar plates (supplemented with 10% OADC). The plates were incubated at 37 °C, and colony-forming units (CFUs) were enumerated after incubation.

### Histochemical and immunofluorescence staining

TRAP staining and immunofluorescence analysis of ST3GAL1 were performed on both murine calvarial specimens and cultured osteoclasts to assess osteoclast presence, maturation, and protein expression. TRAP-positive multinucleated cells (containing ≥3 nuclei) were identified under light microscopy and quantified as osteoclast-lineage cells in randomly selected fields. Fluorescence images were normalized to the control group to ensure accurate comparison of intensity across experimental groups. For immunofluorescence, samples were incubated with a primary antibody against ST3GAL1 (rabbit polyclonal, 1:100), followed by Goat Anti-Rabbit IgG (H + L) Fluor647-conjugated secondary antibody (1:200). Nuclei were counterstained with DAPI, and images were acquired using a confocal fluorescence microscope. Data were analyzed using standardized methods, and fluorescence intensity was normalized relative to the control group for each experiment to ensure the accurate representation of protein expression.

F-actin cytoskeletal organization was assessed in cultured osteoclasts using rhodamine-conjugated phalloidin. Cells were fixed in 4% paraformaldehyde at room temperature for 30 min, permeabilized, and stained according to standard protocols, then visualized by fluorescence microscopy.

All staining procedures were performed under conditions optimized to preserve tissue and cellular integrity. Quantification was performed on raw, unsaturated images with identical acquisition settings; intensity density was background-subtracted and normalized to the control group. Quantitative analyses were conducted across multiple independent experiments to ensure reproducibility.

### MAL II lectin staining

The levels of α2,3-linked sialic acids (α2,3-SA) in both murine calvarial tissue and cultured osteoclasts were assessed using biotinylated Maackia amurensis lectin II (MAL II). Samples were first fixed in 4% paraformaldehyde at room temperature and subsequently washed with PBS, followed by blocking with 1% bovine serum albumin (BSA) for 1 h to minimize nonspecific binding. Biotinylated MAL II was then diluted 1:100 and incubated with the samples overnight at 4 °C to allow specific binding to α2,3-SA residues. For fluorescent visualization, fluorescein-conjugated streptavidin (1:200) was applied at room temperature for 1 h, and nuclei were counterstained with DAPI. All staining procedures were performed under light-protected conditions to prevent photobleaching. Samples were imaged using a confocal fluorescence microscope, and the expression levels and spatial distribution of α2,3-SA in tissue and cells were subsequently analyzed and quantified. Quantification was performed on raw, unsaturated images with identical acquisition settings; intensity density was background-subtracted and normalized to the control group.

### siRNA-mediated gene silencing and quantitative real-time PCR (qRT-PCR)

Three independent siRNAs targeting mouse *St3gal1* were purchased from Hanbio Biotechnology Co., Ltd. and used to knock down gene expression in cultured cells. Cells were seeded at a density of 5 × 10^5^ per well in 6-well plates and transfected with 2 μg of each siRNA using 5 μL of Lipofectamine RNAiMAX (GeneCloud Biotech, Guangzhou) in serum-free Opti-MEM (Gibco), following the manufacturer’s protocol. A Cy3-labeled siRNA targeting *Gapdh* served as a transfection control. Forty-eight hours post-transfection, cells were harvested, and knockdown efficiency was validated at the mRNA level by quantitative real-time PCR (qRT-PCR).

Total RNA was extracted using the RNA-Quick Purification Kit (YiShan Biotech, Shanghai, China) according to the manufacturer’s instructions. RNA concentration and purity were assessed with a NanoDrop ND-1000 spectrophotometer (Thermo Fisher Scientific, Loughborough, UK). A total of 2 µg of RNA was reverse-transcribed into cDNA using a commercial reverse transcription kit (Sangon Biotech, Shanghai, China). qRT-PCR was performed with 1 µL of cDNA, SYBR Green Supermix, and gene-specific primers for *St3gal1* and osteoclast marker genes *Fos, Ocstamp, Mmp9, Nfatc1*, and *CtsK,* with *Gapdh* serving as the internal reference. Amplification was conducted on a QuantStudio 5 Real-Time PCR System (Applied Biosystems) under standard cycling conditions, with all reactions performed in technical triplicates. Relative mRNA expression levels were calculated using the 2^−ΔΔCt^ method, confirming effective gene knockdown and assessing the impact on osteoclast-specific gene expression. Primer sequences are provided in key resources table.

### Western blot analysis

Cells were lysed in RIPA buffer supplemented with protease inhibitors for 30 min on ice to preserve protein integrity. Protein concentrations were determined using a BCA assay, and at least 30 µg of total protein per sample was resolved by 10% SDS-PAGE and subsequently transferred onto PVDF membranes. Membranes were blocked with rapid blocking buffer (Solarbio, Beijing, China) for 15 min at room temperature to minimize nonspecific binding. Following blocking, membranes were incubated overnight at 4 °C with primary antibodies against GAPDH, ST3GAL1 at manufacturer-recommended dilutions. After thorough washing with TBST, membranes were incubated with HRP-conjugated secondary antibodies for 1.5 h at room temperature. Protein bands were detected using an enhanced chemiluminescent (ECL) substrate and visualized with the ChemiDoc Touch Imaging System (Bio-Rad, CA, USA). Densitometric analysis was performed using Image Lab software (Bio-Rad) to quantify relative protein expression levels, with GAPDH serving as the loading control.

### RNA sequencing and analysis

Total RNA was extracted from osteoclasts with or without BCG infection (RB and R groups, n = 3 biological replicates per group)RNA concentration and purity were assessed using a spectrophotometer (Nanodrop 2000), integrity was verified by agarose gel electrophoresis, and RNA integrity number (RIN) was determined using the Agilent 2100 Bioanalyzer. Only samples meeting quality criteria (total RNA ≥1 μg, OD_260/280_ ≥ 1.8, OD_260/230_ ≥ 1.0) were used for library construction. mRNA was enriched from total RNA using poly-T oligo-attached magnetic beads and fragmented to approximately 300 bp. cDNA was synthesized by reverse transcription with random hexamers, followed by second-strand synthesis. Sequencing libraries were constructed using the Illumina TruSeq RNA Sample Prep Kit, with end repair, A-tailing, and adapter ligation, then amplified by PCR. The libraries were subjected to paired-end sequencing (2 × 150 bp) on the Illumina NovaSeq 6000 platform. Raw reads were processed with fastp to remove adapter sequences, poly-N stretches, and low-quality bases. Clean reads were aligned to the mouse reference genome (*Mus musculus* GRCm39, release 109) using HISAT2, and gene-level counts were quantified using featureCounts; FPKM/TPM were calculated for visualization. Differential expression analysis was performed with DESeq2, with significance set at |fold change| > 2 and adjusted P < 0.05. Gene Ontology (GO) and KEGG pathway enrichment of differentially expressed genes was performed using clusterProfiler with the hypergeometric test and FDR correction. Principal component analysis (PCA) and hierarchical clustering were used to assess transcriptome variation across biological replicates and the results were visualized in R (ggplot2).

### Metabolomic profiling and analysis

Metabolomic profiling was performed on osteoclasts from BCG-infected (RB) and uninfected (R) groups (n = 3 biological replicates per group). Cellular metabolites were extracted in 80% methanol containing an internal standard mixture, quenched at −80 °C, centrifuged, and pooled supernatants were used to prepare quality-control (QC) samples. After vacuum drying, extracts were reconstituted in 10% methanol and analyzed by high-resolution LC–MS/MS. Raw data were processed using MS-DIAL for peak detection, alignment, and metabolite annotation against comprehensive databases, including Metlin, MoNA, GNPS, HMDB, and KEGG. Quantitative metabolite matrices were subjected to missing value handling, normalization, and log transformation. Data quality was assessed by the coefficient of variation (CV) of internal standards and by sample-wise Pearson correlation analysis.

Differential metabolites between RB and R were identified primarily using two-sample Student’s t-tests with Benjamini–Hochberg FDR correction (FDR <0.05, together with appropriate fold-change thresholds), with Orthogonal Partial Least Squares Discriminant Analysis (OPLS-DA; ropls R package) and variable importance in projection (VIP >1.0) used as complementary criteria. To elucidate the biological functions and metabolic pathways associated with these changes, Kyoto Encyclopedia of Genes and Genomes (KEGG) pathway enrichment analysis and Metabolite Set Enrichment Analysis (MSEA) were performed using R package corto (version 1.2.4) and visualized in R (ggplot2). For metabolite network analysis, significantly enriched KEGG pathways (typically P < 0.05) and the corresponding pathway-associated differential metabolites were selected to construct a pathway–metabolite regulatory network. Network visualization was performed using the R package ggraph (v2.1.0), with node shapes/colors used to distinguish metabolites and pathways, and metabolite fold changes (FC) encoded in the network display.

### Quantification and statistical analysis

All data were derived from at least three independent experiments, with a minimum biological replicate of n ≥ 3, unless otherwise specified in the figure legends due to sample limitations. Data are presented as mean ± standard deviation (SD), and all error bars in graphical representations correspond to SD. Statistical analyses were performed using GraphPad Prism 8. Comparisons between two groups were conducted using an independent-samples Student’s t-test, while experiments involving three or more groups were analyzed by one-way analysis of variance (ANOVA) followed by Tukey’s *post hoc* test to correct for multiple comparisons. For datasets that did not meet normality assumptions, appropriate non-parametric tests, such as the Mann-Whitney U test or Kruskal–Wallis test, were applied. Statistical significance was defined as *p* < 0.05, with exact *p*-values indicated in the figures.

## Results

### *Mycobacterium tuberculosis* culminates in pathological bone loss via enhanced osteoclast differentiation

**FIGURE 1:**
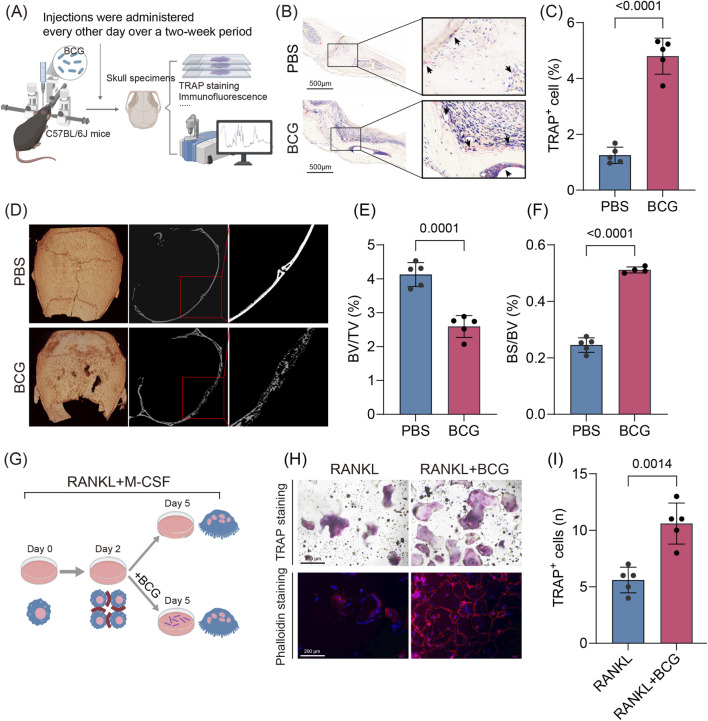
BCG infection promotes osteoclastogenesis and induces bone destruction **(A)** Schematic diagram of a murine cranial model of tuberculous bone destruction induced by BCG infection. **(B,C)** TRAP staining of calvarial sections and quantification of TRAP + multinucleated cells (≥3 nuclei) proportion (n = 5). Proportion was calculated as TRAP^+^ multinucleated cells (≥3 nuclei)/total nucleated cells per field. **(D)** Representative micro-CT images of cranial bone specimens. **(E,F)** Quantitative bone morphometric parameters including BV/TV and BS/BV (n = 5). **(G)** Schematic of *in vitro* osteoclast induction and BCG infection. **(H,I)** Representative TRAP staining and phalloidin staining of cultured osteoclasts and quantification of TRAP^+^ multinucleated cells (≥3 nuclei) per field (randomly selected fields, fixed magnification) *in vitro* (n = 5). Data are presented as mean ± SD. Statistical significance was assessed by two-tailed unpaired Student’s t-test.

Upon invading bone tissue, *M. tuberculosis* (Mtb) triggers aberrant osteoclast activation that culminates in progressive bone destruction, a defining pathological feature of osteoarticular tuberculosis. To recapitulate this process, we established a murine calvarial osteolysis model in which BCG (*Mycobacterium bovis* BCG Pasteur 1173P2) or PBS was administered locally every other day over a 2-week period (Figure 1A). Histological examination revealed a marked accumulation of TRAP-positive multinucleated cells (≥3 nuclei) (Figures 1B,C). Complementary micro-CT reconstruction visualized pronounced cranial bone erosion in BCG-infected mice (Figure 1D). Quantitative morphometric analysis further demonstrated a significant reduction in bone volume fraction (BV/TV, Figure 1E) together with an increased bone surface-to-volume ratio (BS/BV, Figure 1F), reflecting substantial structural deterioration and intensified resorption within the calvaria. To assess the direct effect of mycobacterial infection on osteoclastogenesis, primary bone marrow–derived macrophages (BMMs) were cultured in an *in vitro* osteoclast differentiation system. At 24 h after RANKL stimulation, corresponding to the pre-osteoclast stage, cells were infected with BCG at a multiplicity of infection (MOI) of 10 and co-cultured for an additional 72 h (Figure 1G). Furthermore, the intracellular survival and viability of BCG within the cells were confirmed and quantified via a standard colony-forming unit (CFU) assay (Supplementary Figure S1). TRAP and phalloidin staining demonstrated that BCG infection markedly promoted osteoclast differentiation and maturation, as evidenced by increased numbers of multinucleated TRAP-positive cells and enhanced formation of characteristic F-actin sealing rings (Figures 1H,I). These findings demonstrate that mycobacterial infection robustly accelerates osteoclast activation and function, thereby driving pathological bone destruction.

### α2,3-sialylation contributes to Mtb-induced osteoclastogenesis

**FIGURE 2:**
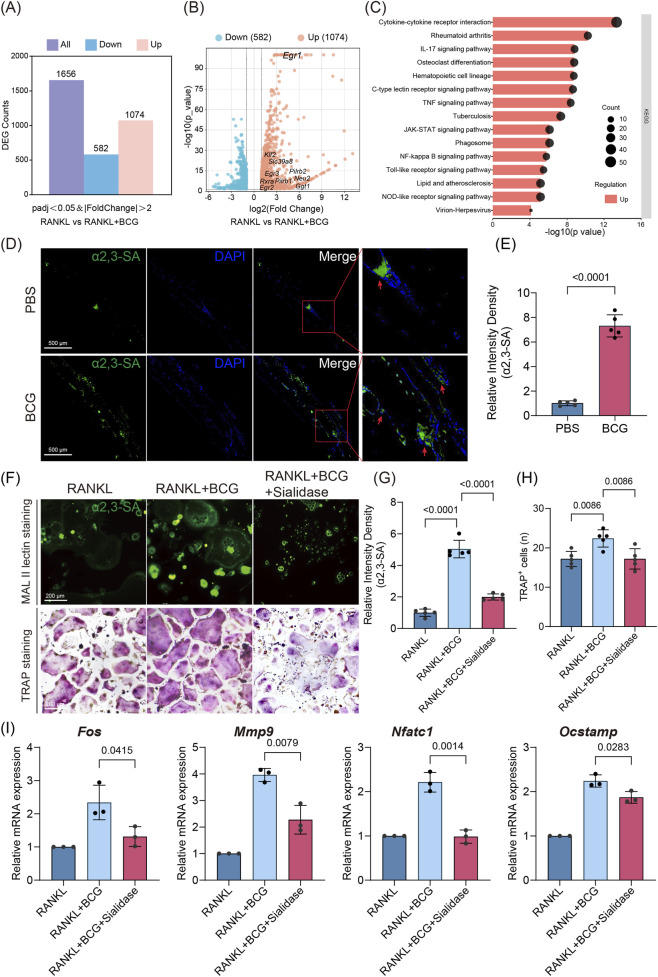
α2,3-sialylation is required for BCG-induced osteoclast differentiation and activity **(A)** Comparison of differentially expressed genes (DEGs) between BCG-infected osteoclasts (RB) and uninfected controls (R). (n = 3 biological replicates per group, differential expression was defined as |FoldChange| > 2 and padj <0.05). **(B)** Volcano plot of DEGs highlighting genes related to sialic acid biosynthesis. **(C)** KEGG pathway enrichment analysis of upregulated DEGs in RB cells. **(D,E)** Immunofluorescence staining of α2,3-SA in mouse calvarial sections using MAL II lectin, with quantification of α2,3-SA fluorescence intensity (n = 5). Intensity density was normalized to the PBS group mean. **(F)** *In vitro* osteoclasts subjected to MAL II lectin staining and TRAP staining, with or without sialidase treatment to enzymatically remove α2,3-SA. **(G)** Quantification of α2,3-SA fluorescence intensity in cultured osteoclasts (n = 5). Intensity density was measured in cellular ROIs after background subtraction and normalized to the RANKL group mean. **(H)** Quantification of TRAP + multinucleated cells (≥3 nuclei) per field (randomly selected fields, fixed magnification) *in vitro* (n = 5). **(I)** mRNA expression levels of osteoclast differentiation markers (*Fos, Mmp9, Nfatc1,* and *Ocstamp*) in osteoclasts (n = 3). Data are presented as mean ± SD. Statistical significance was determined by two-tailed unpaired Student’s t-test for two-group comparisons **(E)** and one-way ANOVA followed by Tukey’s *post hoc* test for three-group comparisons **(G–I)**.

To elucidate the molecular mechanisms underlying mycobacteria-induced osteoclast differentiation, we performed transcriptomic profiling of *in vitro*–differentiated osteoclasts with or without BCG infection. Differential expression analysis revealed that BCG substantially reshaped the osteoclast transcriptome, with 582 genes downregulated and 1,074 genes upregulated (Figure 2A). Emerging evidence indicates that Mtb can modulate host glycosylation to influence cellular functions (Matos et al., 2021). Osteoclasts, derived from monocyte/macrophage lineages, require surface α2,3-sialylation for precursor fusion and maturation into multinucleated bone-resorbing cells. Guided by this, we focused on sialic acid synthesis and metabolism–related genes (Schwarzkopf et al., 2002; Collins et al., 2008; Choi et al., 2024; Wickramasinghe et al., 2011). Notably, transcriptomic profiling identified several significantly upregulated candidates, including *Egr1, Egr2, Egr3, Klf2, Slc39a8, Pilrb1, Pilrb2, Neu2, Rxra*, and *Ggt1*, as visualized in the volcano plot (Figure 2B), suggesting a potential role of sialylation in BCG-enhanced osteoclast differentiation. KEGG pathway enrichment analysis of upregulated genes (Figure 2C) revealed significant enrichment in Osteoclast differentiation, TLR signaling, C-type lectin receptor, JAK-STAT, NF-κB, and IL-17 signaling pathways. Notably, osteoclast differentiation and TLR signaling were strongly activated under Mtb infection, supporting a coordinated transcriptional activation of signaling programs associated with osteoclast differentiation and innate immune responses, aligning with previously reported links between α2,3-sialylation–dependent pathways and osteoclast maturation (Dou et al., 2022).

Building on transcriptomic insights, we next assessed whether Mtb-induced osteoclast hyperactivity correlates with alterations in this specific glycosylation. Immunofluorescence staining of calvarial sections revealed elevated α2,3-SA levels, predominantly localized around resorption lacunae and co-distributed with osteoclasts (Figures 2D,E). *In vitro*, BCG infection similarly increased α2,3-SA levels concomitant with enhanced osteoclast differentiation. Sialidase treatment (10 U/mL) effectively reduced α2,3-SA levels and attenuated the BCG-induced enhancement of osteoclast differentiation (Figures 2F–H). Consistently, mRNA expression of osteoclast markers *Fos, Mmp9, Nfatc1,* and *Ocstamp* mirrored α2,3-SA levels and was markedly suppressed by sialidase treatment (Figure 2I). Together, these results indicate that Mtb infection may promote osteoclast differentiation through upregulation of α2,3-sialylation, providing a potential glycosylation-dependent mechanism underlying tuberculosis-associated bone destruction.

### ST3GAL1 mediates Mtb-induced α2,3-sialylation in osteoclasts

**FIGURE 3:**
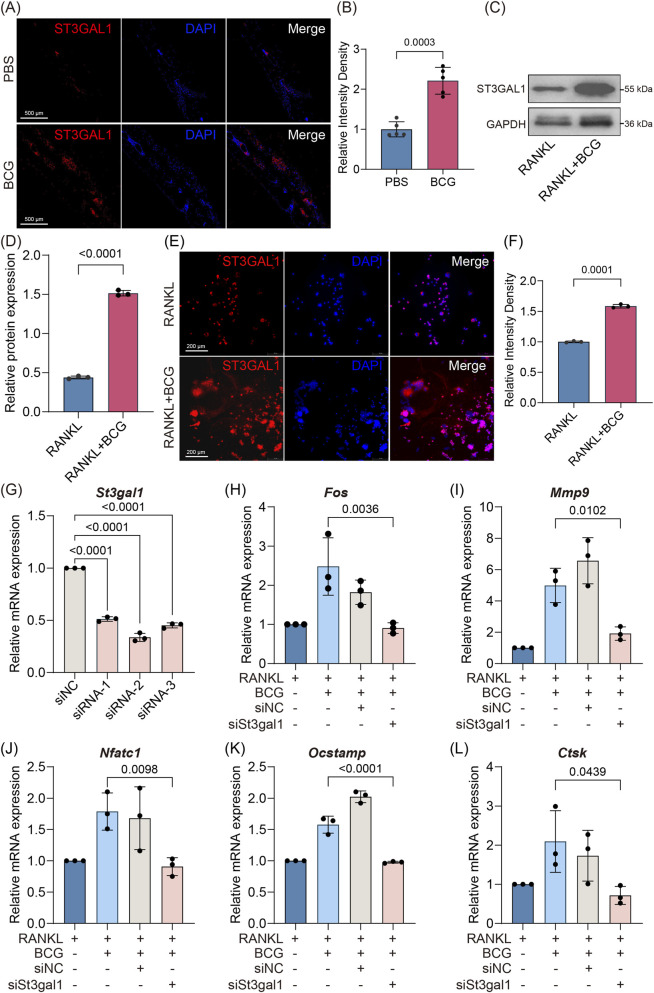
BCG-induced ST3GAL1 upregulation promotes osteoclast differentiation **(A,B)** Representative immunofluorescence images of ST3GAL1 in mouse calvarial sections and corresponding quantification of fluorescence intensity (n = 5). Intensity density was normalized to the PBS group mean. **(C,D)** Western blot analysis of ST3GAL1 protein levels in BCG-infected and uninfected osteoclasts, with corresponding quantification of band intensity (n = 3). **(E,F)** Immunofluorescence staining of ST3GAL1 in cultured osteoclasts and quantification of fluorescence intensity (n = 5). Intensity density was measured in cellular ROIs after background subtraction and normalized to the RANKL group mean. **(G)** Validation of St3gal1 knockdown efficiency at the mRNA level using siRNA (n = 3). **(H–L)** Effects of siSt3gal1 knockdown on mRNA expression of osteoclast differentiation markers (Fos, Mmp9, Nfatc1, Ocstamp and Ctsk) (n = 3). Data are presented as mean ± SD; exact p values are shown in the figure. Comparisons between two groups **(B,D,F)** were performed using unpaired Student’s t-test; multiple group comparisons **(G–L)** were analyzed by one-way ANOVA with Tukey’s *post hoc* test.

Sialylation, defined as the enzymatic addition of sialic acid to acceptor molecules, is primarily regulated by sialyltransferases, among which ST3GAL1 plays a dominant role in mediating α2,3-linked sialylation within the bone microenvironment. Immunofluorescence analysis revealed markedly elevated ST3GAL1 expression in calvarial tissues from BCG-injected mice compared to PBS-treated controls (Figures 3A,B). Consistently, *in vitro* infection of osteoclast precursors with BCG led to robust upregulation of ST3GAL1 protein levels, as confirmed by both Western blotting (Figures 3C,D) and immunofluorescence staining (Figures 3E,F). To elucidate the functional role of ST3GAL1 in Mtb-enhanced α2,3-sialylation, we employed small interfering RNA targeting *St3gal1*. The knockdown efficiency of St3gal1 was confirmed at both the mRNA transcript level via quantitative PCR (Figure 3G) and the protein expression level via Western blotting (Supplementary Figure S2). Silencing *St3gal1* prior to BCG infection substantially suppressed the expression of osteoclastogenic genes, including *Fos, Mmp9, Nfatc1*, *Ocstamp and Ctsk* (Figures 3H–L). Together, these data establish ST3GAL1 as a key enzymatic mediator through which Mtb amplifies pathological osteoclastogenesis via modulation of α2,3-linked sialylation.

### Sialidase alleviates bone destruction induced by Mtb

**FIGURE 4:**
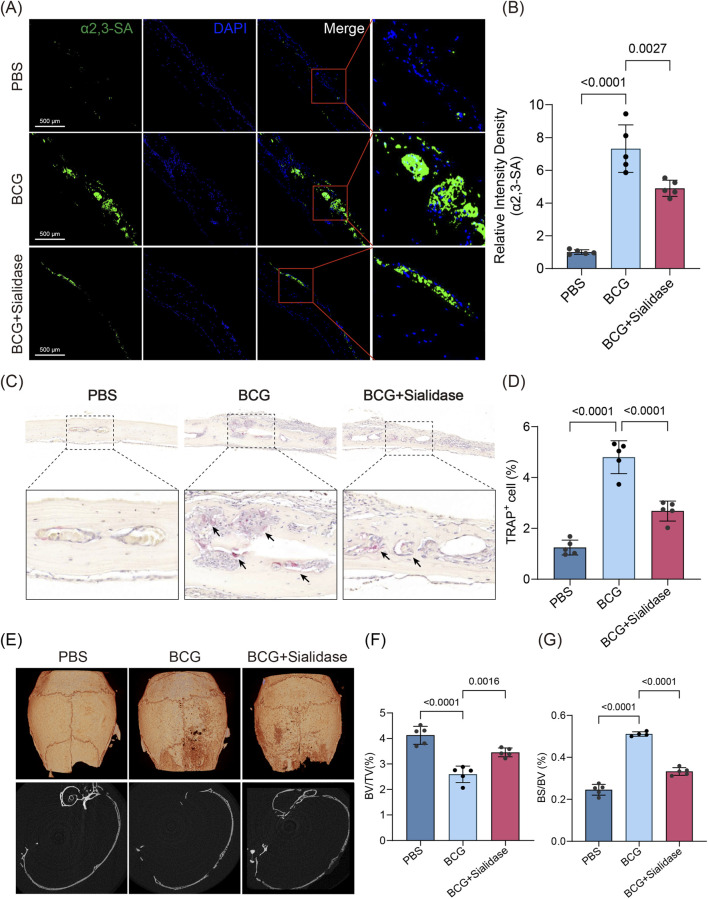
Sialidase treatment attenuates BCG-induced osteoclast activation and bone destruction **(A,B)** Representative immunofluorescence images of α2,3-SA in mouse calvarial sections and corresponding quantification of fluorescence intensity (n = 5). Intensity density was normalized to the PBS group mean. **(C,D)** TRAP staining of calvarial sections and quantification of TRAP^+^ multinucleated cells (≥3 nuclei) proportion (n = 5). Proportion was calculated as TRAP^+^ multinucleated cells (≥3 nuclei)/total nucleated cells per field. **(E–G)** Representative micro-CT images of calvarial bone **(E)** and quantification of bone parameters, including BV/TV (F, n = 5) and BS/BV (G, n = 5). Data are presented as mean ± SD; exact p values are shown in the figure. Comparisons among the three groups were performed using one-way ANOVA followed by Tukey’s multiple comparisons test.

Given the pivotal role of α2,3-SA in Mtb-induced pathological osteoclast activation, we investigated whether targeted desialylation could mitigate infection-mediated bone destruction. Sialidase (SialEXO23, which specifically hydrolyzes α2,3-sialic acids; 10 U/mouse) was administered locally concurrently with BCG calvarial inoculation. Immunofluorescence staining of calvarial sections confirmed the effective hydrolysis of α2,3-SA by sialidase *in vivo* (Figures 4A,B). Subsequent histomorphometric analysis revealed a marked reduction in osteoclast activity within treated lesions, as evidenced by a marked reduction in the prevalence of multinucleated TRAP^+^ cells (Figures 4C,D). Micro-CT scans further demonstrated substantial improvement in bone microarchitecture, characterized by an increased BV/TV and a concomitant reduction in the BS/BV (Figures 4E–G). These results suggest that sialidase treatment effectively attenuates Mtb-induced bone destruction by disrupting pathological α2,3-SA-mediated signaling pathways, highlighting a promising therapeutic strategy to target glycosylation in infection-driven osteolysis.

### Mtb-induced metabolic reprogramming highlights glycerophospholipid-α2,3-SA axis in osteoclast differentiation

**FIGURE 5:**
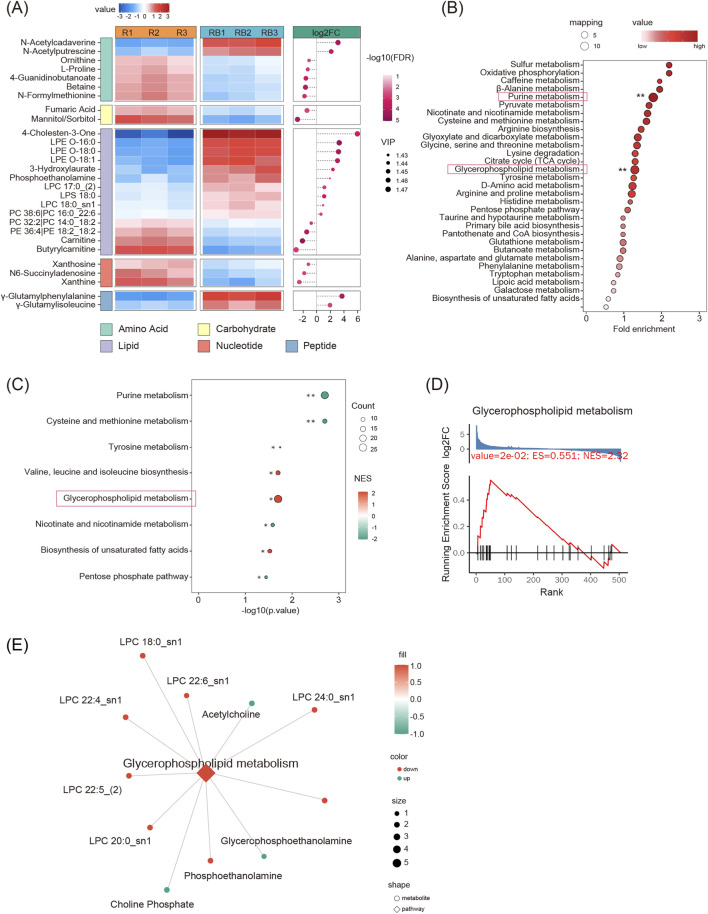
Metabolomic profiling of BCG-infected (RB) versus uninfected (R) osteoclasts. **(A)** Heatmap of differential metabolites across lipid, amino acid, carbohydrate, nucleotide, and small peptide classes, selected by P < 0.05, VIP >1, and |log2FC| > 0.4 (n = 3, Student’s t-test). **(B)** KEGG pathway enrichment of differential metabolites, shown as bubble plots; fold enrichment (log_2_) on the x-axis, circle color represents significance (P < 0.05, Fisher’s exact test), and circle size indicates the number of differential metabolites. **(C)** Metabolite set enrichment analysis (MSEA) by GSEA reveals downregulation of purine metabolism and upregulation of glycerophospholipid metabolism (n = 3, FDR-adjusted). **(D)** Line plot illustrating glycerophospholipid metabolism activation. **(E)** Network of glycerophospholipid metabolism showing differential metabolite regulation (circles: red = up, green = down, size = VIP) and pathway activity (diamonds: red = activated, green = inhibited, size = number of metabolites), indicating enhanced membrane biosynthesis, signaling, and substrate availability for α2,3-SA attachment.

Mtb, an obligate intracellular pathogen, sustains persistent infection by modulating host metabolic pathways (Pandey and Sassetti, 2008; Kaur et al., 2023; Lee et al., 2013). Within the bone microenvironment, such metabolic reprogramming may intersect with osteoclast-specific pathways. Given that α2,3-SA represents a key modification of membrane glycoconjugates, potentially influencing membrane receptors and metabolic transporters (e.g., the SLC family) to directly shape osteoclast metabolic phenotypes—including lipid accumulation and glycolytic reprogramming (Park-Min 2019; Shi et al., 2015; Wang et al., 2024)—we employed metabolomic profiling to systematically assess the metabolic perturbations induced by BCG infection in osteoclasts. By comparing the metabolic pathways of infected and uninfected cells, we aimed to delineate metabolic features associated with elevated α2,3-SA levels and enhanced osteoclast differentiation. Compared with uninfected osteoclasts (R group), BCG-infected osteoclasts (RB group) exhibited a marked enrichment of differential metabolites across lipid, amino acid, carbohydrate, nucleotide, and small peptide classes (Figure 5A). Lipid metabolites, including 4-Cholesten-3-One, LPE O-16:0, and LPE O-18:1, displayed the most pronounced differences, suggesting extensive remodeling of membrane architecture and signaling pathways in the RB group. This remodeling is likely closely associated with alterations in α2,3-sialylated membrane glycoconjugates and membrane receptor functions, representing a potential key driver of enhanced osteoclast differentiation. Amino acid and small peptide metabolites, such as N-Acetylcadaverine, N-Acetylputrescine, Ornithine, and γ-Glutamyl peptides, were broadly upregulated, reflecting enhanced nitrogen metabolism and antioxidative capacity, thereby providing energy and molecular support for cell proliferation and stress responses. Elevated carbohydrate (e.g., Fumaric Acid, Mannitol/Sorbitol) and nucleotide metabolites (e.g., Xanthosine, N6-Succinyladenosine) indicate increased TCA cycle activity, metabolic reprogramming, and augmented nucleotide biosynthesis. The overall upregulation of the RB metabolome reflects BCG-induced metabolic remodeling, implying that α2,3-SA upregulation may coordinate lipid, amino acid, and energy metabolism pathways to synergistically promote osteoclast differentiation and activity.

The KEGG pathway enrichment analysis revealed that differential metabolites were predominantly enriched in purine metabolism and glycerophospholipid metabolism (Figure 5B). Subsequent metabolite set enrichment analysis (MSEA, Figures 5C,D) revealed that, following BCG infection, osteoclasts exhibited an overall downregulation of purine metabolism, whereas glycerophospholipid metabolism was markedly upregulated. Glycerophospholipids, as major constituents of cellular membranes, also provide the biosynthetic foundation for glycosphingolipids, to which α2,3-SA is covalently attached via glycosidic linkages. Network analysis of glycerophospholipid metabolism in the RB group (Figure 5E) showed significant upregulation of key precursors, including acetylcholine, choline phosphate, phosphoethanolamine, and glycerophosphoethanolamine, while multiple lysophosphatidylcholines (LPCs, e.g., LPC 18:0_sn1, LPC 22:4_sn1) were downregulated. These patterns suggest that glycerophospholipid metabolism in RB cells is skewed toward membrane biosynthesis and signal transduction enhancement, with a relative attenuation of phospholipid catabolism, thereby reinforcing membrane integrity, facilitating signaling, and providing additional glycosphingolipid substrates for α2,3-SA attachment. The overall upregulation of glycerophospholipid metabolism not only reflects remodeling of membrane architecture and associated signaling pathways in RB cells but may also directly promote elevated surface α2,3-SA levels, providing crucial metabolic support for enhanced osteoclast differentiation and activity under BCG infection conditions.

Collectively, these metabolomic analyses reveal that BCG infection induces a coordinated remodeling of lipid, amino acid, carbohydrate, and nucleotide metabolism in osteoclasts, with glycerophospholipid metabolism emerging as a key pathway linking membrane biosynthesis, α2,3-SA surface presentation, and enhanced osteoclast differentiation. This integrated metabolic rewiring provides mechanistic insights into how α2,3-SA modulates osteoclast activity under mycobacterial infection, offering a critical foundation for further investigation of targeted metabolic interventions.

## Discussion

This study systematically delineates the molecular basis of pathological osteoclast activation during *Mycobacterium tuberculosis* (Mtb) infection of bone tissue, identifying ST3GAL1-mediated α2,3-sialylation as a critical driver of bone destruction. While prior research in bone tuberculosis has focused primarily on cytokine-mediated mechanisms, our findings demonstrate that Mtb directly hijacks the host glycosylation machinery to potently promote osteoclast differentiation and function, thereby providing a previously unrecognized perspective on bone pathology. Furthermore, untargeted metabolomics uncovered a concurrent reprogramming of host lipid metabolism, specifically an upregulation of glycerophospholipid pathways that may supply essential precursors for α2,3-sialylated membrane conjugates. Although the causal relationship requires further validation, this intriguing observation proposes a potential crosstalk between metabolic homeostasis and post-translational regulation in osteoclasts. Collectively, our work fundamentally advances the understanding of osteoclast-mediated bone pathology in tuberculosis and provides a compelling conceptual framework for therapeutic strategies targeting sialylation pathways.

Although the roles of sialylation in immune regulation—such as ST6GAL-mediated CD22 recognition modulating B cell activation (Fan et al., 2023)—and in tumor metastasis—where elevated α2,3-sialylation is associated with poor prognosis in breast cancer (Park et al., 2024)—have been reported, its function in infectious bone disease remains unexplored. In this study, we provide the first direct evidence linking α2,3-sialylation to aberrant osteoclast activation in tuberculous bone destruction, thereby establishing its pivotal role in Mtb-induced bone pathology. This finding aligns with the emerging concept of “pathogen hijacking host modification pathways,” whereby pathogens manipulate host glycosylation and other post-translational modifications (PTMs) to enhance survival and virulence (Sengupta et al., 2023; Ozdilek et al., 2020). Specifically, in a murine model of tuberculous bone destruction, we observed that BCG infection markedly increased α2,3-sialylation levels in bone tissue. Removal of this modification by sialidase significantly attenuated the ability of Mtb to promote osteoclast activation, indicating that α2,3-sialylation is not merely a passive marker but a critical determinant actively regulating osteoclast fusion and bone-resorptive activity. Consistently, in an *in vitro* osteoclast infection model, silencing of the α2,3-sialyltransferase *St3gal1* substantially suppressed the BCG-induced enhancement of osteoclast differentiation. This resonates with our previous findings (Dou et al., 2022) that ST3GAL1-mediated α2,3-sialylation of TLR2 is essential for osteoclast fusion. Together with prior evidence showing that the Mtb-specific protein Rv1509 directly promotes osteoclast differentiation while impairing osteoblast function via the TLR2 pathway (Liu et al., 2025), these results suggest that TLR2 not only serves as a substrate for α2,3-sialylation but also represents a central signaling node in Mtb-mediated bone destruction. Notably, earlier studies have primarily focused on Mtb’s reliance on intrinsic PTMs to sustain survival and drug tolerance (van Els et al., 2014), such as trehalose metabolism–driven remodeling conferring irreversible resistance (Lee et al., 2019), or O-acetylation contributing to Mtb growth, virulence, and host–pathogen interactions (Birhanu et al., 2017). In contrast, our work provides the first evidence that Mtb actively reprograms host osteoclast α2,3-sialylation to facilitate bone destruction. Our work advances current understanding of interactions between pathogen and host modifications, and highlights host glycosylation pathways as potential therapeutic targets for tuberculous osteomyelitis.

Beyond transcriptional regulation, our metabolomic analysis further revealed a marked reprogramming of glycerophospholipid metabolism in osteoclasts during Mtb infection. This observation raises the possibility of a mechanistic link between α2,3-sialylation and metabolic homeostasis. Glycerophospholipids not only constitute fundamental structural components of cellular membranes but also serve as essential precursors for glycosphingolipid biosynthesis, with glycosphingolipids representing the typical carriers of α2,3-SA modifications (Jeckel et al., 1992). Previous studies in multiple myeloma–associated bone disease have demonstrated that pharmacological blockade of glycosphingolipid synthesis—such as inhibition of GlcCer and LacCer production with GCS inhibitors, or suppression of glucosylceramide synthase using eliglustat—attenuates osteoclast resorptive activity (Leng et al., 2022; Ersek et al., 2015), underscoring the functional connection between lipid metabolism and osteoclast-mediated bone remodeling. In this context, our metabolomic profiling highlights a previously underappreciated possibility: the upregulation of glycerophospholipid metabolism induced by Mtb may represent a metabolic adaptation that enables osteoclasts to sustain their heightened fusion capacity and resorptive activity by providing expanded membrane surfaces and an increased supply of sialylated glyco-conjugates. Specifically, we observed significant accumulation of key intermediates, including phosphoethanolamine and phosphocholine, which could promote the synthesis of glycosphingolipids and other sialylated lipids, thereby facilitating enhanced surface presentation of α2,3-SA in infected osteoclasts. Although isotopic tracing will be required to delineate the precise metabolic flux from lipid precursors to α2,3-sialylation, our findings point toward a new direction for mechanistic investigation.

Although this study systematically delineates the role of α2,3-sialylation in osteoclasts during Mtb infection and its contribution to bone destruction, several limitations warrant consideration. The specific membrane glycoproteins or lipids that undergo pathological α2,3-sialylation in infected osteoclasts remain to be identified. Comprehensive glycoproteomic and glycolipidomic approaches will be essential to define the osteoclast “sialylome” and pinpoint functional targets. In addition, while our metabolomic data suggest that glycerophospholipid reprogramming may provide biosynthetic precursors for α2,3-sialylation, functional validation is required to establish a causal relationship and to determine its precise contribution to osteoclast fusion and resorptive activity. Moreover, although enzymatic desialylation attenuated osteoclast hyperactivation in our murine model, the translational potential of this strategy needs to be assessed in more complex contexts such as chronic bone TB. Future investigations should therefore focus on several directions: systematic identification of key α2,3-sialylated receptors and their downstream fusogenic signals (e.g., via lectin pulldown combined with mass spectrometry); conditional genetic ablation of St3gal1 in osteoclasts to validate its *in vivo* function and contribution to disease progression; pharmacological evaluation of small-molecule ST3GAL1 inhibitors or sialic acid mimetics as potential therapeutic strategies; and exploration of whether this pathway operates more broadly in other infectious or inflammatory bone disorders.

In conclusion, our study identifies α2,3-sialylation as a critical regulator of osteoclast function during bone tuberculosis, underscoring the broader significance of host glycosylation in pathogen-induced bone pathology. While ST3GAL1 serves as a tractable node for modulating this modification, additional glycosylation-related pathways and Mtb-derived factors are likely to contribute to osteoclast activation and bone destruction, warranting further investigation. From a translational standpoint, strategies targeting α2,3-sialylation—such as enzymatic desialylation or small-molecule inhibition—may provide a complementary approach to conventional anti-tuberculosis therapy to mitigate osteolytic damage and improve clinical outcomes. More broadly, exploring host-directed therapies that modulate glycosylation or osteoclast activity could expand the therapeutic landscape for bone tuberculosis. By extending the conceptual framework of bone tuberculosis beyond inflammatory cytokine networks to encompass osteoclast regulation and glycosylation dynamics, this work offers a foundation for integrating host-targeted interventions with standard bone tuberculosis chemotherapy to optimize patient care.

## Data Availability

The datasets presented in this study can be found in online repositories. The names of the repository/repositories and accession number(s) can be found below: https://www.ncbi.nlm.nih.gov/, PRJNA1314431 https://www.ebi.ac.uk/metabolights/, MTBLS12992.
