# Supplementary material for: Mycobacterium tuberculosis infection drives osteoclast overactivation via α2,3-Sialylation to promote pathological bone destruction
Source: Front Pharmacol. 2026 Apr 2;17:1738896. doi: 10.3389/fphar.2026.1738896 (PMC13084157; doi:10.3389/fphar.2026.1738896)
Supplement: Supplementary file 1 [file DataSheet1.pdf]

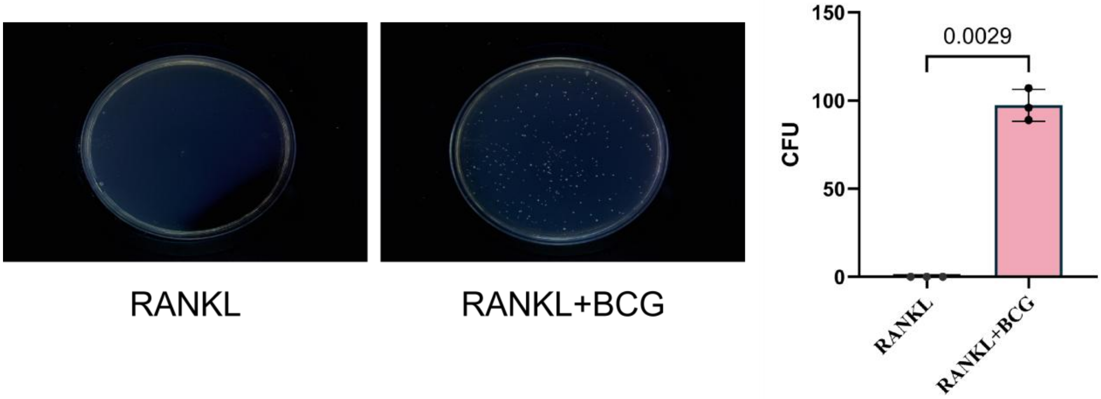

Supplementary Figure S1. Assessment of intracellular BCG viability. Intracellular survival of Mycobacterium bovis BCG within the infected cells was quantified using a standard colony-forming unit (CFU) assay. Data are presented as mean ± SD (n = 3).

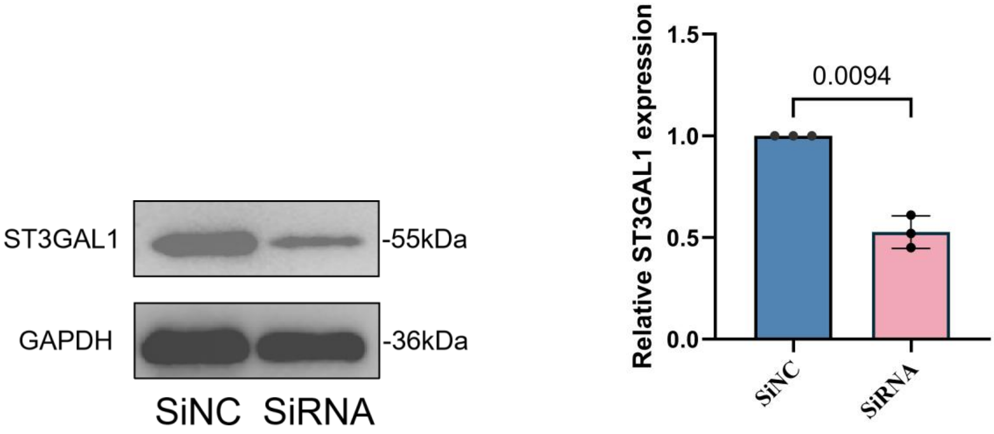

Supplementary Figure S2. Validation of *St3gal1* knockdown efficiency at the protein level. Western blot analysis of ST3GAL1 protein expression in cells transfected with *St3gal1*-specific siRNA compared to negative control (NC) siRNA.
